# Analysis of the interventional effects of ambroxol hydrochloride and ontelukast sodium combined with azithromycin on immune function and C-reactive protein serum expression in children with severe Mycoplasma pneumoniae pneumonia

**DOI:** 10.3389/fped.2026.1698182

**Published:** 2026-08-03

**Authors:** Xueru Liu, Xiang Chen, Shan Ye

**Affiliations:** 1 Department of Respiratory Medicine, Wuhan Children’s Hospital, Tongji Medical College, Huazhong University of Science & Technology, Wuhan, Hubei, China; 2 Department of Pulmonary and Critical Care Medicine, The Sixth Hospital of Wuhan, Affiliated Hospital of Jianghan University, Wuhan, Hubei, China; 3 Department of Pediatrics, Changde Hospital, Xiangya School of Medicine, Central South University (The First people’s Hospital of Changde City), Changde, Hunan, China

**Keywords:** ambroxol hydrochloride, azithromycin, C-Reactive protein, immune function, montelukast sodium, severe Mycoplasma pneumonia

## Abstract

**Objective:**

The aim of the study was to investigate the clinical effects of ambroxol hydrochloride and montelukast sodium combined with azithromycin on immune function, inflammatory cytokines, and pulmonary function in children with severe Mycoplasma pneumoniae pneumonia (SMPP).

**Methods:**

A retrospective cohort study was conducted on 103 children with SMPP treated at Wuhan Children's Hospital, Tongji Medical College, Huazhong University of Science & Technology, from February 2023 to February 2025. On the basis of different treatment regimens recorded in the electronic medical record system, the children were divided into a control group (*n* = 50, montelukast sodium combined with azithromycin) and an observation group (*n* = 53, ambroxol hydrochloride added to the control regimen). To address the selection bias inherent in the retrospective design, a propensity score analysis based on inverse probability of treatment weighting (IPTW) was performed; a logistic regression model incorporating age, sex, disease duration, only-child status, paternal and maternal education levels, and place of residence as covariates was used to derive the propensity scores, and stabilized weights were applied so that all 103 patients contributed to the weighted analysis without exclusion. Clinical data were collected and compared, including baseline characteristics, symptoms and signs (time for cough to subside, time for body temperature to normalize, time for sputum to disappear, and time for rales to disappear), immune function (CD3+, CD4+, CD8+, CD4+/CD8+), inflammatory cytokines [tumor necrosis factor-alpha (TNF-α), interferon-gamma (IFN-γ), interleukin-2 (IL-2), interleukin-4 (IL-4), and C-reactive protein (CRP)], pulmonary function [forced expiratory volume in the first second (FEV1), forced vital capacity (FVC), and maximal mid-expiratory flow (MMEF)], clinical efficacy, and adverse reactions. The total clinical efficacy rate was pre-specified as the primary outcome, and all remaining endpoints were treated as secondary or exploratory; a Bonferroni-corrected significance threshold of *α* = 0.003 was applied across the 16 secondary continuous outcomes. Effect sizes (Cohen's *d*) and 95% confidence intervals (CIs) were computed for all between-group comparisons.

**Results:**

After treatment, the observation group showed significantly greater improvement in symptoms and signs, immune function, inflammatory cytokines, and pulmonary function compared with the control group (all *P* < 0.05). The total clinical efficacy rate of the observation group was significantly higher than that of the control group [96.23% vs. 82.00%; *χ*^2^ = 5.459, *P* = 0.019; odds ratio (OR) = 5.59, 95% CI: 1.14–27.38; the wide confidence interval, driven by the small number of ineffective cases, indicates that the magnitude of the efficacy advantage should be interpreted with caution]. The total incidence of adverse reactions was comparable between the observation group and the control group (11.32% vs. 10.00%; *χ*^2^ = 0.047, *P* = 0.828), indicating acceptable safety.

**Conclusion:**

The triple combination of ambroxol hydrochloride, montelukast sodium, and azithromycin is associated with improved inflammatory status, enhanced immune function, and better pulmonary function in children with SMPP, with a favorable safety profile. These findings warrant confirmation in prospective, multicenter randomized controlled trials.

## 1 Introduction

Mycoplasma pneumoniae is one of the smallest self-replicating pathogenic microorganisms that occupies an intermediate position between viruses and bacteria; it lacks a cell wall and is capable of passing through bacterial filters (1, 2). Mycoplasma pneumoniae pneumonia (MPP) is a respiratory disease caused by this pathogen and represents a leading cause of community-acquired pneumonia in children (3, 4). In recent years, the incidence of severe Mycoplasma pneumoniae pneumonia (SMPP) has increased markedly in pediatric clinical settings. Garzoni et al. (5) reported that, in addition to the typical respiratory manifestations, children with SMPP frequently present with persistent hyperthermia, extrapulmonary complications, and intractable airway hyperresponsiveness. The underlying pathologic mechanism is closely linked to immune dysregulation and an excessive inflammatory response. Accumulating evidence indicates that Mycoplasma pneumoniae infection triggers a Th1/Th2 immune imbalance, characterized by a decreased CD4+/CD8+ ratio, abnormal immunoglobulin secretion, and activation of the leukotriene pathway, which collectively promote a marked elevation in inflammatory markers such as C-reactive protein (CRP), potentially culminating in a “cytokine storm” that exacerbates pulmonary tissue damage (6, 7). Therefore, the identification of scientifically sound and effective clinical treatment strategies is of considerable importance.

Azithromycin is the first-line treatment for pediatric Mycoplasma pneumoniae pneumonia (MPP). As a macrolide antibiotic, it effectively inhibits pathogen replication; however, its immunomodulatory and anti-inflammatory capacity is limited, underscoring the need to explore multi-targeted combination approaches (8, 9). Leukotrienes are key inflammatory mediators in the progression of pneumonia that stimulate airway smooth muscle contraction, increase vascular permeability, and promote chemotaxis of inflammatory cells, leading to increased respiratory secretions, heightened airway responsiveness, and reduced pulmonary function (10). Montelukast sodium, a selective cysteinyl leukotriene receptor antagonist, attenuates airway spasm and reduces secretions by blocking the binding of cysteinyl leukotrienes (CysLTs) to their receptors, and additionally promotes airway ciliary clearance and sputum discharge, thereby improving lung ventilation (11). Chen et al. (12) demonstrated that the combination of montelukast sodium and azithromycin improved pulmonary function indices such as FEV1 and FVC and restored the CD4+/CD8+ ratio toward normal levels in children. Ambroxol hydrochloride is a widely used mucolytic and expectorant agent that not only reduces sputum viscosity and promotes the synthesis of alveolar surfactant but also enhances bronchial ciliary motility and exerts antioxidant and anti-inflammatory effects (13, 14). Relevant studies have confirmed that ambroxol hydrochloride can elevate IgA and IgG levels while simultaneously reducing serum CRP in children with MPP (15, 16). However, existing literature has focused predominantly on two-drug combination regimens, and the synergistic mechanism and clinical benefit of triple therapy with ambroxol hydrochloride, montelukast sodium, and azithromycin in SMPP have not been systematically evaluated.

On this basis, the present study was designed to investigate the clinical value of the triple combination of ambroxol hydrochloride, montelukast sodium, and azithromycin in children with SMPP, with the aim of comprehensively assessing its regulatory effects on immune function, inflammatory cytokines, and pulmonary function, clarifying its relationship with immune reconstitution, and providing an objective evidence base for the development of individualized clinical dosing regimens.

## 2 Materials and methods

### 2.1 Ethical statement

This study was approved by the Ethics Committee of Wuhan Children's Hospital, Tongji Medical College, Huazhong University of Science & Technology (Approval No.: 2024HWH1102). All research procedures were conducted in strict accordance with the Declaration of Helsinki. Given the retrospective nature of the study and the exclusive use of de-identified data, the Ethics Committee waived the requirement for individual informed consent, in accordance with applicable regulations and ethical guidelines for retrospective studies.

### 2.2 Study design and sample size estimation

This was a retrospective cohort study. An *a priori* sample size calculation was performed using G*Power 3.1.9.7 software. Based on a two-tailed independent-samples *t*-test with a significance level of *α* = 0.05, a statistical power of 1 − *β* = 0.80, and an anticipated medium-to-large effect size of Cohen's *d* = 0.60 [derived from prior studies on ambroxol hydrochloride combination therapy in pediatric pneumonia (15, 16)], the minimum required sample size was calculated to be 45 patients per group (total *N* = 90). Accounting for an anticipated data attrition rate of approximately 10%, the target enrollment was set at 50 patients per group. A total of 103 children with SMPP treated at Wuhan Children's Hospital between February 2023 and February 2025 were ultimately included.

### 2.3 Propensity score weighting and group allocation

Because this study was retrospective in nature, a propensity score analysis based on inverse probability of treatment weighting (IPTW) was employed to reduce potential selection bias, following the framework described by Austin and Stuart (21). A multivariable logistic regression model was constructed with group assignment (observation vs. control) as the dependent variable and the following pre-specified covariates: age, sex, disease duration, only-child status, paternal education level, maternal education level, and place of residence. The C-statistic of the propensity score model was 0.62, indicating moderate but acceptable separation, and the propensity score distributions of the two groups demonstrated adequate overlap with no observations falling outside the region of common support. Stabilized inverse probability weights were derived as *w* = ($\hat{P}$(*T* = 1))/(*ê*(*X*)) for treated patients and *w* = ($\hat{P}$(*T* = 0))/(*1* − *ê*(*X*)) for controls, where *ê*(*X*) denotes the estimated propensity score; the resulting weights ranged from 0.71 to 1.46 with a mean of 1.00, and no extreme weights were truncated. After weighting, the effective sample sizes were 50.8 in the observation group and 47.6 in the control group, and absolute standardized mean differences for all measured baseline covariates were below 0.10, indicating satisfactory covariate balance. All 103 patients were retained in the analysis without exclusion, and weighted means and proportions were used for between-group comparisons; sensitivity analyses using overlap weights produced results that were qualitatively unchanged from the IPTW estimates, supporting the robustness of the primary findings. The observation group comprised 53 patients (ambroxol hydrochloride, montelukast sodium, and azithromycin), and the control group comprised 50 patients (montelukast sodium and azithromycin).

### 2.4 Inclusion and exclusion criteria

The inclusion criteria were as follows: (1) diagnosis of SMPP based on the criteria established in the Expert Consensus on Laboratory Diagnosis and Clinical Practice of Mycoplasma Pneumoniae Infection in Children in China (2019 edition) (17), defined as MPP accompanied by any of the following: persistent fever exceeding 38.5 °C for more than 5 days despite appropriate macrolide therapy, radiographic evidence of large lobar consolidation or bilateral involvement, extrapulmonary complications, or significant hypoxemia (SpO_2_ < 92% on room air); (2) presence of clinical symptoms including cough and fever; (3) positive Mycoplasma pneumoniae culture from pharyngeal secretions or positive serum Mycoplasma pneumoniae IgM antibody titer; (4) absence of congenital diseases; and (5) no known allergy to the study medications.

The exclusion criteria were as follows: (1) coexisting severe anemia (hemoglobin < 60 g/L); (2) presence of hepatic or renal dysfunction (alanine aminotransferase > 2× upper limit of normal or serum creatinine above age-specific reference range); (3) altered consciousness; (4) intolerance to any of the study drugs; and (5) concurrent asthma, allergic rhinitis, congenital pulmonary malformation, or other active infectious diseases.

### 2.5 Treatment protocol

Both groups received standard supportive care, including appropriate bed positioning, ward disinfection and ventilation, and antipyretic therapy as clinically indicated (physical cooling for body temperature 37.5–38.5 °C and acetaminophen or ibuprofen for temperature >38.5 °C). Children in the control group received azithromycin dry suspension (Pfizer Pharmaceutical Co., Ltd.; National Drug Approval No. H10960112; specification: 0.1 g per sachet) administered orally at 10 mg/kg on the first day (once daily) followed by 5 mg/kg on days 2 through 5 (once daily), combined with chewable montelukast sodium tablets (Sichuan Otsuka Pharmaceutical Co., Ltd.; National Drug Approval No. H20064828; specification: 5 mg) at a dose of 5 mg once daily, taken orally after meals.

In the observation group, ambroxol hydrochloride injection (Chengdu Bite Pharmaceutical Co., Ltd.; National Drug Approval No. H20183196; specification: 2 mL:15 mg) was administered in addition to the control regimen. A dose of 7.5–15 mg of ambroxol hydrochloride (adjusted according to body weight: 7.5 mg for children weighing <20 kg and 15 mg for those weighing ≥20 kg) was diluted in 100 mL of 5% glucose solution and administered by slow intravenous infusion twice daily for 7 consecutive days. The doses of azithromycin and montelukast sodium were identical to those in the control group.

All children were treated for a total of 2 weeks. Clinical status was monitored closely throughout the treatment period. Clinically relevant indices were collected at two time points: before the first treatment and after the completion of the full treatment course.

### 2.6 Data collection

Baseline data were extracted from the electronic medical record system and included age, sex (male/female), disease duration, only-child status (yes/no), paternal education level (junior high school and below/senior high school and above), maternal education level (junior high school and below/senior high school and above), and place of residence (urban/rural).

### 2.7 Assessment of symptoms and signs

The following clinical endpoints were recorded: time for cough to subside (defined as resolution of cough frequency to ≤2 episodes per day), time for body temperature to normalize (defined as axillary temperature ≤37.2 °C for ≥24 consecutive hours without antipyretics), time for sputum to disappear (defined as absence of visible sputum production), and time for rales to disappear (defined as absence of rales on bilateral chest auscultation).

### 2.8 Assessment of immune function

Immune function was evaluated by measuring CD3+, CD4+, and CD8+ T-lymphocyte percentages and the CD4+/CD8+ ratio. Peripheral venous blood samples (5 mL) were collected from the antecubital vein under fasting conditions in the morning, transferred into ethylenediaminetetraacetic acid (EDTA)-anticoagulated tubes, and processed within 2 h of collection. Samples were centrifuged at 3000 r/min for 10 min using a centrifuge (model: LL900; Luoyang Hongshi Machinery Equipment Co., Ltd.), and the separated serum was stored at −80 °C until analysis. T-lymphocyte subsets were determined using a flow cytometer (FACSCalibur; BD Biosciences, San Jose, CA, USA) with fluorochrome-conjugated monoclonal antibodies (CD3-FITC, CD4-PE, CD8-APC; BD Biosciences), following the manufacturer's protocol. Quality control was performed with calibration beads supplied by the manufacturer before each analytical run.

### 2.9 Assessment of inflammatory cytokines

Serum concentrations of TNF-α, IFN-γ, IL-2, IL-4, and CRP were determined. Peripheral venous blood samples were collected, centrifuged, and stored as described in Section 2.8. TNF-α, IFN-γ, IL-2, and IL-4 were quantified using commercial enzyme-linked immunosorbent assay (ELISA) kits (Boster Biological Technology Co., Ltd., Wuhan, China) with intra-assay and inter-assay coefficients of variation of <8% and <12%, respectively. CRP was measured using an immunoturbidimetric method on an automated biochemistry analyzer (Hitachi 7600; Hitachi, Tokyo, Japan) with a lower limit of detection of 0.1 mg/L. All assays were performed in duplicate, and the mean values were used for analysis.

### 2.10 Assessment of pulmonary function

Pulmonary function was assessed using a spirometer (PowerCube-Body; GANSHORN Medizin Electronic GmbH, Niederlauer, Germany) calibrated daily according to the manufacturer's specifications. Measurements included forced expiratory volume in the first second (FEV1), forced vital capacity (FVC), and maximal mid-expiratory flow (MMEF). All values were expressed as percentages of predicted values for age, height, and sex. Each child performed at least three acceptable maneuvers, and the best values were recorded in accordance with American Thoracic Society/European Respiratory Society (ATS/ERS) standards (18).

### 2.11 Assessment of clinical efficacy

Clinical efficacy was evaluated with reference to the Expert Consensus on the Diagnosis and Treatment of Macrolide-Resistant Mycoplasma Pneumoniae Pneumonia in Children (19) and classified into three categories. Clinical cure was defined as complete resolution of fever, cough, sputum production, and pulmonary rales, with clear bilateral lung fields on chest radiography, normalization of complete blood count, and a negative Mycoplasma pneumoniae antibody test. Marked improvement was defined as substantial improvement of the above clinical symptoms and signs, radiographic evidence of inflammatory absorption with residual changes, normalization of complete blood count, and a negative Mycoplasma pneumoniae antibody test. Ineffectiveness was defined as no improvement in clinical symptoms, signs, imaging findings, or laboratory parameters. The total effective rate was calculated as: (number of clinical cure cases + number of marked improvement cases)/total number of cases × 100%.

### 2.12 Assessment of adverse reactions

Adverse reactions were monitored throughout the treatment period and categorized as dizziness, headache, nausea, or gastrointestinal discomfort. The severity of each adverse reaction was graded according to the Common Terminology Criteria for Adverse Events version 5.0 (CTCAE v5.0) (20). The time of onset, duration, management measures, and outcome of each adverse event were documented. The total incidence of adverse reactions was compared between the two groups.

### 2.13 Statistical methods

Statistical analyses were performed using SPSS 25.0 (IBM Corp., Armonk, NY, USA). The normality of continuous variables was assessed using the Shapiro–Wilk test, and homogeneity of variances was evaluated by Levene's test. Normally distributed continuous data are expressed as mean ± standard deviation ($\bar{x}\pm s$) and compared between groups using the independent-samples *t*-test under IPTW; within-group comparisons across time points were performed using the paired-samples *t*-test. Non-normally distributed data were analyzed using the Mann–Whitney *U* test. Categorical data are presented as counts and percentages (*n*, %) and analyzed using the chi-square test or Fisher's exact test as appropriate. Two complementary analyses were applied to the three-category clinical efficacy outcome to align the analytical approach with the data structure: the ordinal distribution of cure, marked improvement, and ineffective categories was compared between groups using the Mann–Whitney *U* test, and the binary collapsed outcome of total effective rate (cure plus marked improvement vs. ineffective) was assessed using the chi-square test with the corresponding odds ratio (OR) and 95% CI. The total effective rate was pre-specified as the single primary outcome of the study, and all 16 continuous secondary outcomes (four symptom-resolution times, four immune-function indices, five inflammatory cytokines, and three pulmonary-function indices) were analyzed under a Bonferroni-corrected significance threshold of *α* = 0.05/16 = 0.003 to control the family-wise error rate; outcomes with *P*-values between 0.003 and 0.05 are reported as exploratory. The total incidence of adverse reactions was treated as a co-primary safety endpoint at *α* = 0.05. Effect sizes for continuous outcomes were calculated as Cohen's *d* (with values of 0.20, 0.50, and 0.80 representing small, medium, and large effects, respectively), and 95% confidence intervals (CIs) for between-group mean differences were computed. For the primary outcome (total clinical efficacy), the odds ratio (OR) and its 95% CI were calculated. A complete *post hoc* power calculation was conducted for every continuous outcome using G * Power 3.1.9.7 with *α* = 0.05 (two-tailed), *n*1 = 53, and *n*2 = 50, on the basis of the observed Cohen's *d*. All statistical tests were two-tailed, and a *P*-value of <0.05 was considered statistically significant.

## 3 Results

### 3.1 Comparison of baseline characteristics

Comparison of age, sex, disease duration, only-child status, paternal education level, maternal education level, and place of residence between the observation group and the control group revealed no statistically significant differences (all *P* > 0.05), confirming comparability at baseline (Table 1). The absolute standardized mean differences for all measured covariates were below 0.10 after IPTW, indicating adequate covariate balance in the weighted sample.

**Table 1:** Comparison of baseline characteristics between the two groups of children with severe Mycoplasma pneumoniae pneumonia (SMPP).

Index	Project	Observation group (*n* = 53)	Control group (*n* = 50)	*χ*^2^/*t*	*P*
Age (years, $\bar{x}\pm s$)		7.34 ± 1.35	7.62 ± 1.28	1.079	0.283
Sex (*n*, %)	Male	36 (67.92)	30 (60.00)	0.702	0.402
	Female	17 (32.08)	20 (40.00)		
Disease duration (d, $\bar{x}\pm s$)		4.27 ± 1.57	4.35 ± 1.66	0.251	0.802
Only child (*n*, %)	Yes	29 (54.72)	32 (64.00)	0.918	0.338
	No	24 (45.28)	18 (36.00)		
Paternal education (*n*, %)	Junior high school and below	20 (37.74)	17 (34.00)	0.156	0.693
	Senior high school and above	33 (62.26)	33 (66.00)		
Maternal education (n, %)	Junior high school and below	17 (32.08)	22 (44.00)	1.555	0.212
	Senior high school and above	36 (67.92)	28 (56.00)		
Place of residence (n, %)	Urban	35 (66.04)	31 (62.00)	0.089	0.766
	Rural	19 (33.96)	19 (38.00)		

### 3.2 Comparison of symptoms and signs

As shown in Table 2, the observation group demonstrated significantly shorter times for cough to subside, body temperature to normalize, sputum to disappear, and rales to disappear compared with the control group (all *P* < 0.001). The mean between-group differences and associated effect sizes were as follows: cough subsidence [mean difference (MD) = 3.76 d, 95% CI: 2.97–4.55, Cohen's *d* = 1.85], body temperature normalization (MD = 2.47 d, 95% CI: 1.74–3.20, Cohen's *d* = 1.31), sputum disappearance (MD = 3.50 d, 95% CI: 2.16–4.84, Cohen's *d* = 1.02), and rales disappearance (MD = 4.27 d, 95% CI: 3.39–5.15, Cohen's *d* = 1.89). These findings indicate that the addition of ambroxol hydrochloride to montelukast sodium and azithromycin is associated with significantly faster resolution of clinical symptoms and signs in children with SMPP.

**Table 2:** Comparison of symptoms and signs between the two groups (d, $\bar{x}\pm s$).

Index	Observation group (*n* = 53)	Control group (*n* = 50)	MD (95% CI)	t	*P*	Cohen's *d*
Cough subsidence	10.55 ± 1.89	14.31 ± 2.17	3.76 (2.97, 4.55)	9.392	<0.001	1.85
Temperature normalization	5.11 ± 1.84	7.58 ± 1.92	2.47 (1.74, 3.20)	6.667	<0.001	1.31
Sputum disappearance	11.56 ± 3.27	15.06 ± 3.57	3.50 (2.16, 4.84)	5.193	<0.001	1.02
Rales disappearance	12.58 ± 2.22	16.85 ± 2.30	4.27 (3.39, 5.15)	9.587	<0.001	1.89

MD, mean difference; CI, confidence interval.

### 3.3 Comparison of immune function

Before treatment, there were no statistically significant differences in CD3+, CD4+, CD8+, or CD4+/CD8+ between the observation group and the control group (all *P* > 0.05), as shown in Table 3.

**Table 3:** Comparison of immune function before treatment ($\bar{x}\pm s$).

Index	Observation group (*n* = 53)	Control group (*n* = 50)	*t*	*P*
CD3+ (%)	51.74 ± 1.62	51.65 ± 1.55	0.288	0.774
CD4+ (%)	33.25 ± 1.62	33.14 ± 1.57	0.350	0.727
CD8+ (%)	47.02 ± 3.71	46.95 ± 3.65	0.096	0.923
CD4+/CD8+	0.71 ± 0.11	0.73 ± 0.14	0.809	0.421

**Figure 1:**
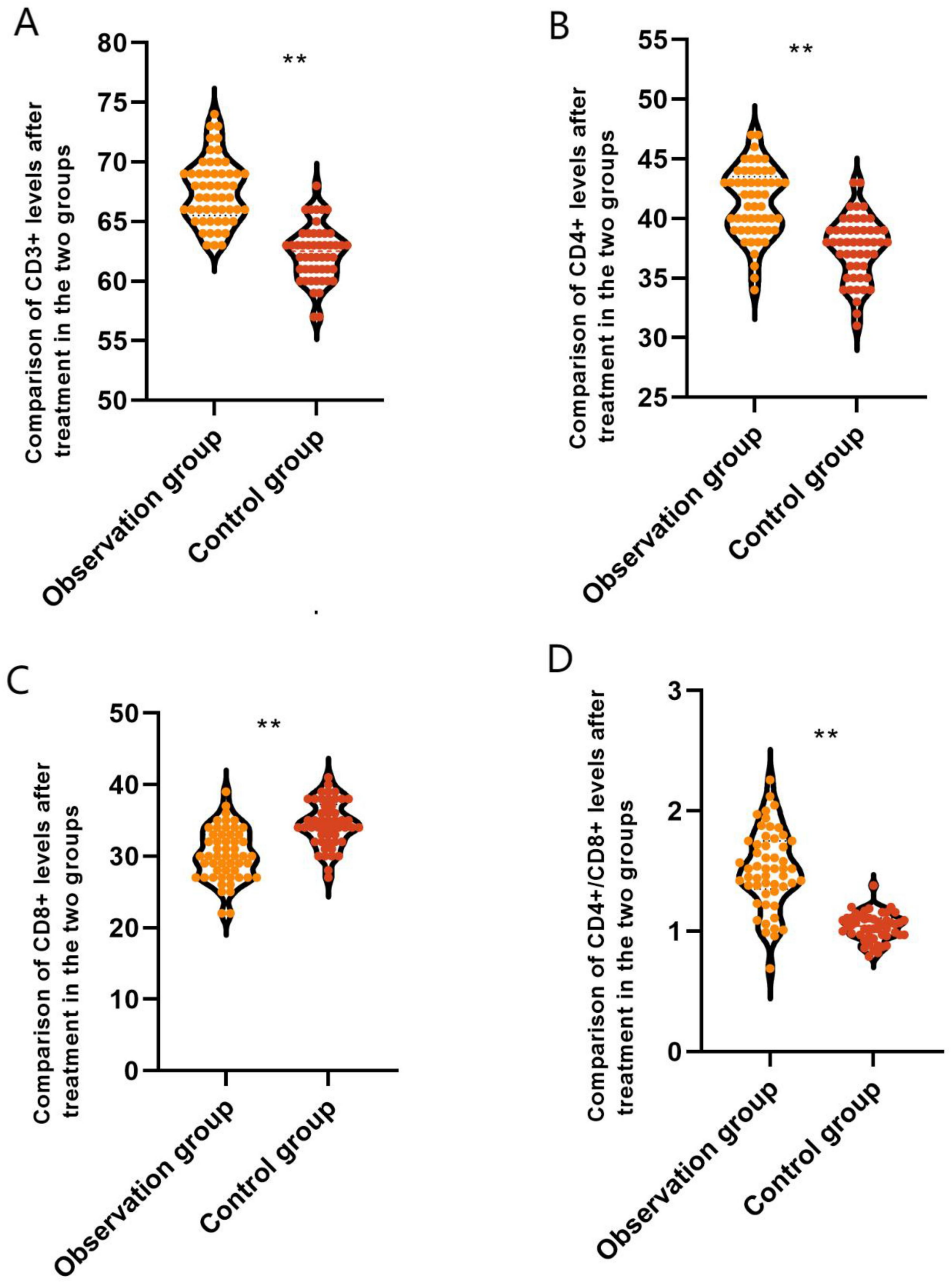
Comparison of immune function indicators between the two groups after treatment. Panel **A**: CD3+ (%), Panel **B**: CD4+ (%), Panel **C**: CD8+ (%), Panel D: CD4+/CD8+ ratio. Data are presented as mean ± standard deviation. Observation group (*n* = 53): ambroxol hydrochloride + montelukast sodium + azithromycin; Control group (*n* = 50): montelukast sodium + azithromycin. Between-group comparisons were performed using independent samples *t*-tests. *P < 0.05; **P < 0.001.

After treatment, the observation group exhibited significantly higher levels of CD3+, CD4+, and CD4+/CD8+ and significantly lower levels of CD8+ compared with the control group (all *P* < 0.05). The between-group differences were as follows: CD3+ (MD = 5.22%, 95% CI: 4.24–6.20, Cohen's *d* = 2.06), CD4+ (MD = 3.84%, 95% CI: 2.74–4.94, Cohen's *d* = 1.37), CD8+ (MD = −4.30%, 95% CI: −5.63 to −2.97, Cohen's *d* = 1.26), and CD4+/CD8+ (MD = 0.48, 95% CI: 0.41–0.55, Cohen's *d* = 1.99). These results indicate that the triple combination regimen is associated with more effective restoration of cellular immune function in children with SMPP (Table 3A, Figure 1).

**Table 3A:** Comparison of immune function after treatment ($\bar{x}\pm s$).

Index	Observation group (*n* = 53)	Control group (*n* = 50)	*t*	*P*
CD3+ (%)	65.42 ± 2.51	60.20 ± 2.55	10.470	<0.001
CD4+ (%)	42.18 ± 2.78	38.34 ± 2.83	6.946	<0.001
CD8+ (%)	38.95 ± 3.38	43.25 ± 3.45	6.385	<0.001
CD4+/CD8+	1.18 ± 0.24	0.70 ± 0.24	10.107	<0.001

### 3.4 Comparison of inflammatory cytokines

Before treatment, no statistically significant differences were observed in TNF-α, IFN-γ, IL-2, IL-4, or CRP between the two groups (all *P* > 0.05), as shown in Table 4.

**Table 4:** Comparison of inflammatory cytokines before treatment ($\bar{x}\pm s$).

Index	Observation group (*n* = 53)	Control group (*n* = 50)	*t*	*P*
TNF-α (ng/mL)	15.11 ± 4.71	14.99 ± 4.85	0.127	0.899
IFN-γ (ng/mL)	26.23 ± 5.47	26.25 ± 5.50	0.018	0.985
IL-2 (ng/mL)	304.25 ± 51.69	305.62 ± 53.14	0.133	0.895
IL-4 (ng/mL)	2.26 ± 0.78	2.28 ± 0.85	0.125	0.901
CRP (mg/L)	57.88 ± 10.67	58.15 ± 9.87	0.133	0.894

TNF-α, tumor necrosis factor-alpha; IFN-γ, interferon-gamma; IL-2, interleukin-2; IL-4, interleukin-4; CRP, C-reactive protein.

**Figure 2:**
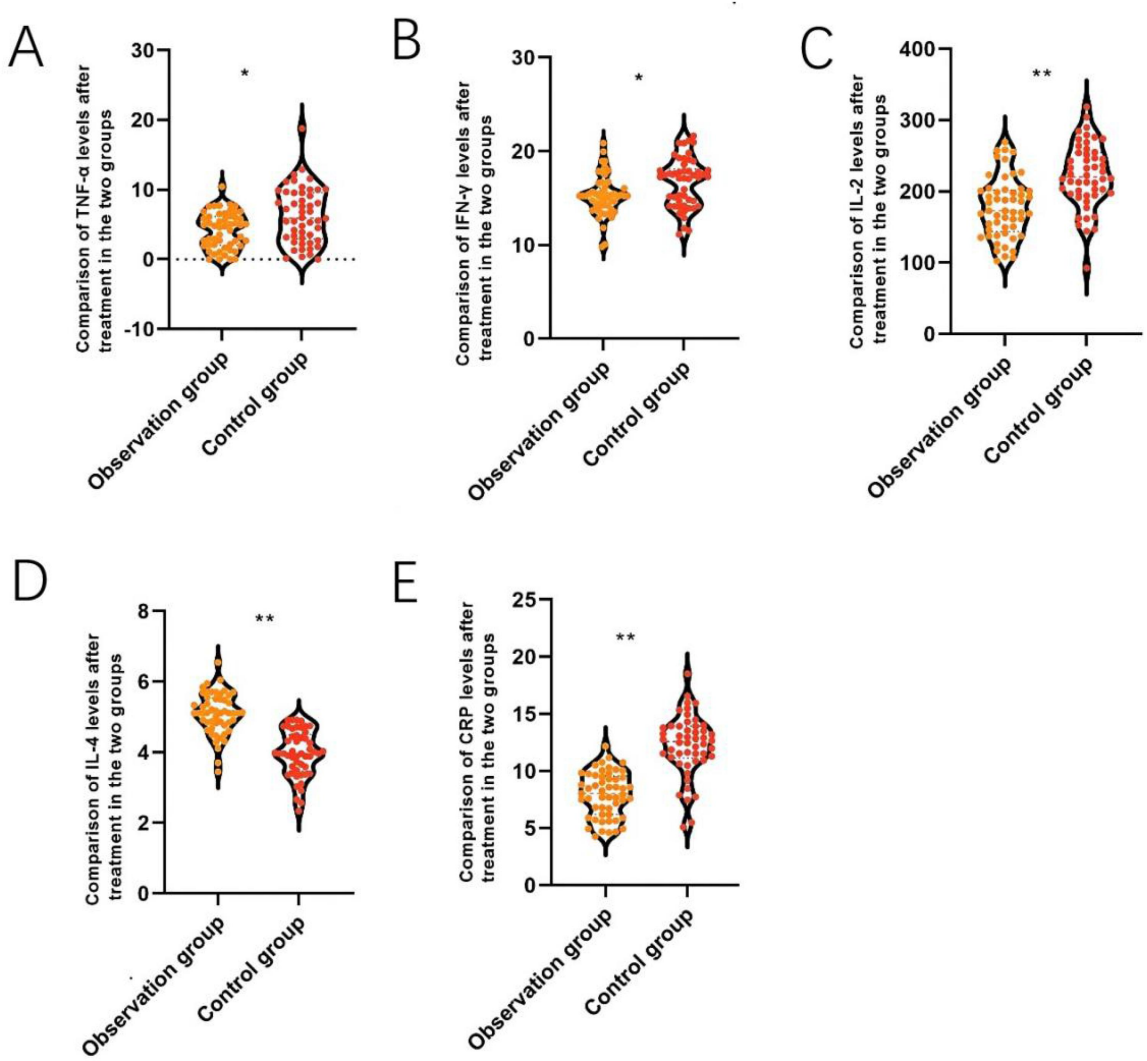
Comparison of inflammatory cytokine indicators between the two groups after treatment. Panel **A**: TNF-α (ng/mL), Panel **B**: IFN-γ (ng/mL), Panel **C**: IL-2 (ng/mL), Panel **D**: IL-4 (ng/mL), Panel **E**: CRP (mg/L). Data are presented as mean ± standard deviation. Observation group (*n* = 53): ambroxol hydrochloride + montelukast sodium + azithromycin; Control group (*n* = 50): montelukast sodium + azithromycin. Between-group comparisons were performed using independent-samples *t*-tests. TNF-α, tumor necrosis factor-alpha; IFN-γ, interferon-gamma; IL-2, interleukin-2; IL-4, interleukin-4; CRP, C-reactive protein. *P < 0.05; **P < 0.001.

After treatment, the observation group showed significantly lower levels of TNF-α, IFN-γ, IL-2, and CRP and significantly higher levels of IL-4 compared with the control group (all *P* < 0.05). The between-group comparisons were: TNF-α (MD = −2.16 ng/mL, 95% CI: −3.49 to −0.83, Cohen's *d* = 0.64), IFN-γ (MD = −1.40 ng/mL, 95% CI: −2.34 to −0.46, Cohen's *d* = 0.58), IL-2 (MD = −44.78 ng/mL, 95% CI: −61.96 to −27.60, Cohen's *d* = 1.02), IL-4 (MD = 1.15 ng/mL, 95% CI: 0.91–1.39, Cohen's *d* = 1.87), and CRP (MD = −4.29 mg/L, 95% CI: −5.21 to −3.37, Cohen's *d* = 1.84). These findings suggest that the addition of ambroxol hydrochloride is associated with more effective modulation of the inflammatory response in children with SMPP (Table 4A, Figure 2).

**Table 4A:** Comparison of inflammatory cytokines after treatment ($\bar{x}\pm s$).

Index	Observation group (*n* = 53)	Control group (*n* = 50)	*t*	*P*
TNF-α (ng/mL)	8.45 ± 3.30	10.61 ± 3.46	3.247	0.002
IFN-γ (ng/mL)	18.12 ± 2.39	19.52 ± 2.43	2.945	0.004
IL-2 (ng/mL)	198.45 ± 42.34	243.23 ± 45.20	5.187	<0.001
IL-4 (ng/mL)	4.18 ± 0.61	3.03 ± 0.62	9.490	<0.001
CRP (mg/L)	12.34 ± 2.30	16.63 ± 2.36	9.331	<0.001

TNF-α, tumor necrosis factor-alpha; IFN-γ, interferon-gamma; IL-2, interleukin-2; IL-4, interleukin-4; CRP, C-reactive protein.

### 3.5 Comparison of pulmonary function

Before treatment, no statistically significant differences in FEV1, FVC, or MMEF were observed between the two groups (all *P* > 0.05), as shown in Table 5.

**Table 5:** Comparison of pulmonary function before treatment (% predicted, $\bar{x}\pm s$).

Index	Observation group (*n* = 53)	Control group (*n* = 50)	*t*	*P*
FEV1	43.56 ± 3.71	43.89 ± 3.94	0.438	0.662
FVC	44.89 ± 2.78	45.39 ± 2.21	1.007	0.317
MMEF	48.73 ± 3.13	49.01 ± 2.97	0.465	0.643

FEV1, forced expiratory volume in the first second; FVC, forced vital capacity; MMEF, maximal mid-expiratory flow.

**Figure 3:**
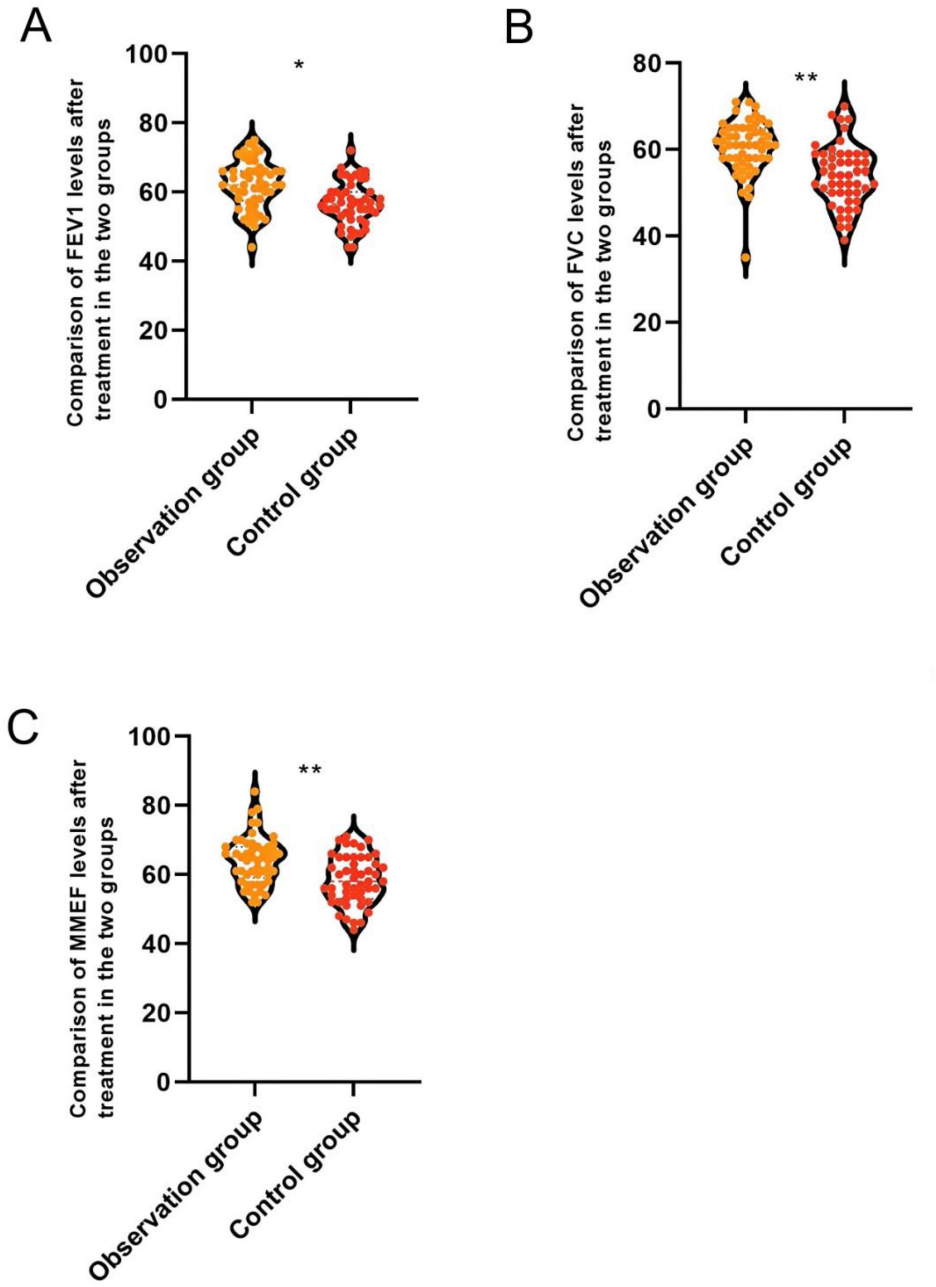
Comparison of pulmonary function indicators between the two groups after treatment. Panel **A**: FEV1 (% predicted), Panel **B**: FVC (% predicted), Panel **C**: MMEF (% predicted). Data are presented as mean ± standard deviation. Observation group (*n* = 53): ambroxol hydrochloride + montelukast sodium + azithromycin; Control group (*n* = 50): montelukast sodium + azithromycin. Between-group comparisons were performed using independent-samples *t*-tests. FEV1, forced expiratory volume in the first second; FVC, forced vital capacity; MMEF, maximal mid-expiratory flow. *P < 0.05; **P < 0.001.

After treatment, the observation group demonstrated significantly higher levels of FEV1, FVC, and MMEF compared with the control group (all *P* < 0.05). The between-group differences were as follows: FEV1 (MD = 5.49%, 95% CI: 2.90–8.08, Cohen's *d* = 0.83), FVC (MD = 6.41%, 95% CI: 3.80–9.02, Cohen's *d* = 0.96), and MMEF (MD = 5.76%, 95% CI: 3.02–8.50, Cohen's *d* = 0.82). These findings indicate that the triple therapy regimen is associated with superior improvement in pulmonary function in children with SMPP (Table 5A, Figure 3).

**Table 5A:** Comparison of pulmonary function after treatment (% predicted, $\bar{x}\pm s$).

Index	Observation group (*n* = 53)	Control group (*n* = 50)	*t*	*P*
FEV1 (%)	76.56 ± 6.66	71.07 ± 6.55	4.211	<0.001
FVC (%)	78.65 ± 6.74	72.24 ± 6.59	4.873	<0.001
MMEF (%)	77.34 ± 7.07	71.58 ± 6.96	4.155	<0.001

FEV1, forced expiratory volume in the first second; FVC, forced vital capacity; MMEF, maximal mid-expiratory flow.

### 3.6 Comparison of clinical efficacy

The total clinical efficacy rate of the observation group (96.23%) was significantly higher than that of the control group (82.00%; *χ*^2^ = 5.459, *P* = 0.019; OR = 5.59, 95% CI: 1.14–27.38). The wide confidence interval reflects limited precision arising from the small number of ineffective cases in the observation group (*n* = 2) and indicates that, although the direction of the effect is consistent, the magnitude of the efficacy advantage should be interpreted with caution. As a complementary analysis aligned with the ordinal nature of the three-category outcome, a Mann–Whitney *U* test on the ranked distribution of clinical cure, marked improvement, and ineffective categories also showed a statistically significant between-group difference favoring the observation group (*Z* = 2.398, *P* = 0.016), corroborating the chi-square finding under both analytical perspectives. The clinical cure rate was 39.62% in the observation group vs. 26.00% in the control group, and the marked improvement rate was 56.60% vs. 56.00%, respectively. These results indicate that the addition of ambroxol hydrochloride to montelukast sodium and azithromycin is associated with higher clinical efficacy in the treatment of SMPP in children (Table 6).

**Table 6:** Comparison of clinical efficacy between the two groups (*n*, %).

Index	Observation group (*n* = 53)	Control group (*n* = 50)	*χ*^2^/*Z*	*P*
Clinical cure	21 (39.62)	13 (26.00)	–	–
Marked improvement	30 (56.60)	28 (56.00)	–	–
Ineffective	2 (3.77)	9 (18.00)	–	–
Total effective rate	51 (96.23)	41 (82.00)	5.459	0.019

Odds ratio (OR) for total efficacy (observation vs. control) = 5.59, 95% confidence interval (CI): 1.14–27.38.

### 3.7 Comparison of adverse reactions

The total incidence of adverse reactions was 11.32% (6/53) in the observation group and 10.00% (5/50) in the control group, with no statistically significant difference (*χ*^2^ = 0.047, *P* = 0.828; OR = 1.15, 95% CI: 0.33–4.01). All observed adverse events were classified as Grade 1 according to CTCAE v5.0 criteria and resolved spontaneously without discontinuation of treatment or specific medical intervention. The median duration of adverse events was 1.5 days (interquartile range: 1.0–2.5 days) in the observation group and 2.0 days (interquartile range: 1.0–3.0 days) in the control group. No serious adverse events (Grade ≥3) occurred in either group during the study period (Table 7).

**Table 7:** Comparison of adverse reactions between the two groups (*n*, %).

Index	Observation group (*n* = 53)	Control group (*n* = 50)	*χ* ^2^	*P*
Dizziness	2 (3.77)	1 (2.00)	–	–
Headache	1 (1.89)	2 (4.00)	–	–
Nausea	2 (3.77)	1 (2.00)	–	–
Gastrointestinal discomfort	1 (1.89)	1 (2.00)	–	–
Total incidence	6 (11.32)	5 (10.00)	0.047	0.828

All adverse events were Grade 1 per Common Terminology Criteria for Adverse Events version 5.0 (CTCAE v5.0). Odds ratio (OR) for total adverse reactions (observation vs. control) = 1.15, 95% confidence interval (CI): 0.33–4.01.

### 3.8 *Post hoc* statistical power assessment

A complete *post hoc* power calculation was performed for every continuous outcome on the basis of the observed Cohen's *d*, fixed sample sizes (*n*1 = 53, *n*2 = 50), and a two-tailed *α* of 0.05, using G * Power 3.1.9.7. Achieved power exceeded 0.95 for all symptom-resolution, immune-function, and pulmonary-function indices, as well as for the inflammatory cytokines IL-2, IL-4, and CRP. Among the inflammatory cytokines, TNF-α had an achieved power of 0.895, while IFN-γ had an achieved power of 0.830, which is the lowest value observed across the panel and approaches but does not reach the conventional threshold of 0.80 with a comfortable margin; this finding is consistent with the smaller observed effect size for IFN-γ (Cohen's *d* = 0.58) and indicates that the IFN-γ result should be interpreted with proportionate caution. The complete *post hoc* power profile for all 16 continuous outcomes is summarized in Table 8.

**Table 8:** *Post hoc* statistical power for all continuous outcomes (*n*1 = 53, *n*2 = 50, α = 0.05, two-tailed).

Outcome	Cohen's *d*	Achieved power	Domain	Interpretation
Cough subsidence	1.85	>0.999	Symptoms	Adequate
Temperature normalization	1.31	>0.999	Symptoms	Adequate
Sputum disappearance	1.02	>0.999	Symptoms	Adequate
Rales disappearance	1.89	>0.999	Symptoms	Adequate
CD3+	2.06	>0.999	Immune	Adequate
CD4+	1.37	>0.999	Immune	Adequate
CD8+	1.26	>0.999	Immune	Adequate
CD4+/CD8+	1.99	>0.999	Immune	Adequate
TNF-α	0.64	0.895	Cytokines	Adequate
IFN-γ	0.58	0.830	Cytokines	Borderline
IL-2	1.02	>0.999	Cytokines	Adequate
IL-4	1.87	>0.999	Cytokines	Adequate
CRP	1.84	>0.999	Cytokines	Adequate
FEV1	0.83	0.986	Pulmonary	Adequate
FVC	0.96	0.998	Pulmonary	Adequate
MMEF	0.82	0.985	Pulmonary	Adequate

CD3+, Cluster of differentiation 3 positive (T cells); CD4+, Cluster of differentiation 4 positive (helper T cells); CD8+, Cluster of differentiation 8 positive (cytotoxic T cells); CD4+/CD8+, Ratio of helper T cells to cytotoxic T cells; TNF-α, Tumor necrosis factor-alpha (inflammatory cytokine); IFN-γ, Interferon-gamma (antiviral cytokine); IL-2, Interleukin-2 (T cell growth factor); IL-4, Interleukin-4 (anti-inflammatory cytokine); CRP, C-reactive protein (inflammation marker); FEV1, Forced expiratory volume in 1 s (lung function test); FVC, Forced vital capacity (lung function test); MMEF, Maximal mid-expiratory flow (lung function test).

## 4 Discussion

SMPP is a common and potentially severe respiratory disease in toddlers and school-age children, characterized by rapid onset and the potential for serious complications, including emphysema and pulmonary atelectasis if not treated promptly (22, 23). Clinical manifestations are diverse, with cough as the predominant symptom, and the principal therapeutic objectives include cough suppression, adequate oxygenation, and relief of bronchospasm (24). Although macrolide antibiotics remain the mainstay of treatment owing to their favorable pharmacokinetic properties, including good oral bioavailability, broad tissue distribution, acid stability, and prolonged half-life, single-agent therapy is often insufficient for optimal outcomes, particularly in severe cases (8, 9).

T-lymphocyte subsets play a central role in the immune response to Mycoplasma pneumoniae infection. CD3+ serves as a marker of mature T lymphocytes and reflects overall cellular immune function. CD4+ T helper cells regulate the immune response by recognizing antigens and secreting cytokines that activate B cells and macrophages. CD8+ cytotoxic T lymphocytes directly kill virally infected or tumor cells and exert immunosuppressive effects. The CD4+/CD8+ ratio reflects the state of immune balance and is a key indicator of immune homeostasis (25, 26). Among inflammatory cytokines, TNF-α regulates immune responses and induces inflammation and apoptosis. IFN-γ activates macrophage phagocytosis and enhances the antiviral immune response. IL-2 promotes T-cell proliferation and differentiation and enhances natural killer cell activity. IL-4 drives Th2-cell differentiation, suppresses the release of proinflammatory factors, and regulates allergic responses. CRP is an acute-phase protein synthesized by the liver whose elevation reflects systemic inflammation (27, 28). FEV1, FVC, and MMEF are established indicators of pulmonary ventilatory function and are important for evaluating the severity and recovery of lung involvement in SMPP (29).

In the pathogenesis of SMPP, Mycoplasma pneumoniae adhesion proteins disrupt the epithelial barrier, while bacterial toxins induce apoptosis. The resultant Th2-dominant immune response suppresses cellular immunity, and autoantibodies can attack pulmonary tissue and activate the complement system through the classical pathway, promoting increased vascular permeability, inflammatory exudation, airway structural disruption, and mucus plug formation (30). These pathological processes collectively result in abnormalities in inflammatory cytokines, immune indicators, and pulmonary function.

The present study demonstrated that both groups showed improvement after treatment, confirming the therapeutic benefit of montelukast sodium combined with azithromycin, consistent with the findings of Gong et al. (31). Azithromycin is a semi-synthetic 15-membered macrolide antibiotic with a stable chemical structure and strong anti-infective activity. Its ability to achieve intracellular concentrations substantially higher than serum levels allows effective bactericidal activity against intracellular Mycoplasma pneumoniae (8). However, azithromycin alone has limited capacity to suppress non-specific inflammation. The addition of montelukast sodium, a selective leukotriene receptor antagonist, addresses this limitation by blocking leukotriene release from mast cells and eosinophils, reducing vascular permeability and bronchospasm, and alleviating tracheal mucosal edema, thereby improving airway inflammatory responses and pulmonary ventilation function (32, 33). This complementary mechanism of action provides the rationale for the two-drug combination; however, further enhancement of clinical outcomes through triple therapy is worth exploring.

Ambroxol hydrochloride is a metabolite of bromhexine and a widely used mucolytic and expectorant agent. Its primary pharmacological actions include degradation of sputum glycoproteins to reduce viscosity, promotion of alveolar surfactant synthesis and secretion by type II pneumocytes, enhancement of bronchial ciliary motility to facilitate sputum clearance, and reduction of respiratory resistance (13, 14). In addition, ambroxol hydrochloride possesses significant antioxidant properties, effectively scavenging oxygen-free radicals and inhibiting phospholipase A2, thereby reducing arachidonic acid release and attenuating allergic and inflammatory responses (34, 35). Importantly, ambroxol hydrochloride has been shown to directly act on the glands of the airway mucosa, increasing the local concentration of concurrently administered antimicrobial agents within airway secretions, which may potentiate the bactericidal effect of azithromycin (15).

In the present study, the observation group demonstrated superior improvement across all outcome domains (symptoms and signs, immune function, inflammatory cytokines, and pulmonary function) compared with the control group, with all effect sizes being medium to large (Cohen's *d* range: 0.58–2.06). The total clinical efficacy rate was significantly higher in the observation group (96.23% vs. 82.00%; OR = 5.59, 95% CI: 1.14–27.38; *P* = 0.019). These results suggest that the addition of ambroxol hydrochloride to the two-drug regimen may confer synergistic benefit through multiple mechanisms. With respect to immune modulation, ambroxol hydrochloride has been reported to inhibit mast cell degranulation and histamine release, suppress Th2 cytokine overproduction, reduce the inhibitory effect of regulatory T cells within the inflammatory microenvironment, and promote IFN-γ-mediated Th1 immune responses, thereby ameliorating the Th1/Th2 imbalance (36, 37). Its capacity to block the NF-*κ*B signaling pathway further enhances macrophage phagocytic activity and inhibits neutrophil-mediated peroxidation, contributing to reduced inflammatory mediator damage (36). These immunomodulatory and anti-inflammatory properties, combined with its mucolytic, surfactant-promoting, and antibiotic-potentiating effects, likely account for the superior outcomes observed in the triple therapy group.

Regarding safety, the total incidence of adverse reactions did not differ significantly between the observation group and the control group (11.32% vs. 10.00%; *P* = 0.828). All adverse events were mild (CTCAE Grade 1), self-limiting, and did not necessitate treatment discontinuation or additional intervention. The median duration of adverse events was comparable between groups. No serious adverse events (Grade ≥ 3) were observed throughout the study period. These findings suggest that the addition of ambroxol hydrochloride to the two-drug regimen does not substantially increase the risk of adverse events and that the triple combination has an acceptable safety profile.

Several limitations of this study should be acknowledged. First, the retrospective design is inherently susceptible to selection bias and unmeasured confounding. Although IPTW was applied to balance measured covariates between groups and produced absolute standardised mean differences below 0.10 for all baseline variables, the moderate C-statistic of 0.62 indicates only moderate separation in the propensity score distributions, and residual confounding from unmeasured variables (such as pre-treatment antibiotic exposure, severity of radiographic findings, and nutritional status) cannot be excluded. Second, the relatively small sample size (*n* = 103) limits the statistical power to detect small effect sizes and restricts the generalizability of the findings. A complete *post hoc* power calculation across all 16 continuous outcomes confirmed achieved power above 0.95 for all symptom-resolution, immune-function, and pulmonary-function endpoints, while the inflammatory cytokines TNF-α and IFN-γ had achieved powers of 0.895 and 0.830, respectively, the IFN-γ result remaining the lowest in the panel and warranting cautious interpretation given its smaller observed effect size (Cohen's *d* = 0.58). Although the primary outcome of total clinical efficacy reached statistical significance with OR = 5.59 (95% CI: 1.14–27.38), the wide confidence interval—driven by only two ineffective cases in the observation group—indicates that the magnitude of the efficacy advantage is estimated with limited precision; the lower bound of the interval, while excluding unity, is close to the null and the point estimate should not be regarded as a stable estimate of the true effect size. Third, this was a single-center study conducted at one institution, which may limit the external validity and reproducibility of the results due to the homogeneity of the patient population. Fourth, the 2-week treatment and follow-up period may be insufficient to capture long-term outcomes and late-onset adverse events. Fifth, the clinical efficacy assessment relied on a composite categorical outcome without a validated scoring system, and the ordinal nature of the efficacy grades may not fully capture the continuous spectrum of clinical improvement. Because the study was retrospective and non-blinded, ascertainment bias in the assignment of cure, marked improvement, and ineffective categories cannot be excluded; the operational definitions adopted from the relevant Chinese expert consensus involved subjective clinical judgements that were not pre-specified through a validated, reproducible scoring instrument, and treating physicians were aware of the regimen received. Sixth, the two regimens differed in route of administration, with ambroxol hydrochloride delivered by intravenous infusion in the observation group while the control regimen was administered exclusively orally; this asymmetry may have contributed to differential intensity of clinical monitoring and to expectation effects in non-blinded outcome ascertainment, and constitutes a potential source of performance bias that future prospective studies should address through identical routes of administration or through blinded outcome adjudication. Seventh, despite the application of a Bonferroni-corrected significance threshold of *α* = 0.003 across the 16 continuous secondary outcomes, the multiplicity inherent in evaluating outcomes across four functional domains may still inflate the family-wise type I error; the secondary findings should therefore be interpreted as hypothesis-generating and supportive of the primary efficacy result rather than independently confirmatory. Future prospective, multicenter, randomized controlled trials with larger sample sizes, longer follow-up periods, and standardized outcome measures are needed to confirm and extend these findings.

## 5 Conclusion

In summary, the triple combination of ambroxol hydrochloride, montelukast sodium, and azithromycin is associated with superior clinical outcomes in children with SMPP compared with dual therapy. The triple regimen was associated with more rapid symptom resolution, enhanced immune function, reduced inflammatory cytokine levels, and improved pulmonary function, without a significant increase in adverse events. These findings support the potential clinical utility of this triple therapy approach and provide a basis for further investigation in well-designed prospective trials.

## Data Availability

The original contributions presented in the study are included in the article/Supplementary Material, further inquiries can be directed to the corresponding author.
